# Supplementary material for: Physical Characterization and In Vitro Toxicity Test of PDMS Synthesized from Low-Grade D4 Monomer as a Vitreous Substitute in the Human Eyes
Source: J Funct Biomater. 2022 Jan 2;13(1):3. doi: 10.3390/jfb13010003 (PMC8788430; doi:10.3390/jfb13010003)
Supplement: Supplementary file 1 [file jfb-13-00003-s001.zip › jfb-1492241-SI.pdf]

Physical Characterization and In Vitro Toxicity Test of PDMS Synthesized from Low Grade D4 Monomer as A Vitreous Substitute in the Human Eyes

Diba Grace Auliya [1,*], Soni Setiadji [2,3], Fitrilawati Fitrilawati [1] and Risdiana Risdiana [1,*]

[1] Department of Physics, Faculty of Mathematics and Natural Sciences, Universitas Padjadjaran, Jl. Raya Bandung-Sumedang km 21 Jatinangor, Sumedang 45363, Indonesia; fitrilawati@phys.unpad.ac.id (FF)
[2] Department of Chemistry, Faculty of Mathematics and Natural Sciences, Universitas Padjadjaran, Jl. Raya Bandung-Sumedang km 21 Jatinangor, Sumedang 45363, Indonesia; s.setiadji@uinsgd.ac.id (SS)
[3] Department of Chemistry, Faculty of Sciences and Technology, UIN Sunan Gunung Djati Bandung, Jl. A.H. Nasution No. 105 Cibiru, Bandung 40614, Indonesia
* Correspondence: diba15001@mail.unpad.ac.id (DGA); risdiana@phys.unpad.ac.id (RR)

(a)
(b)

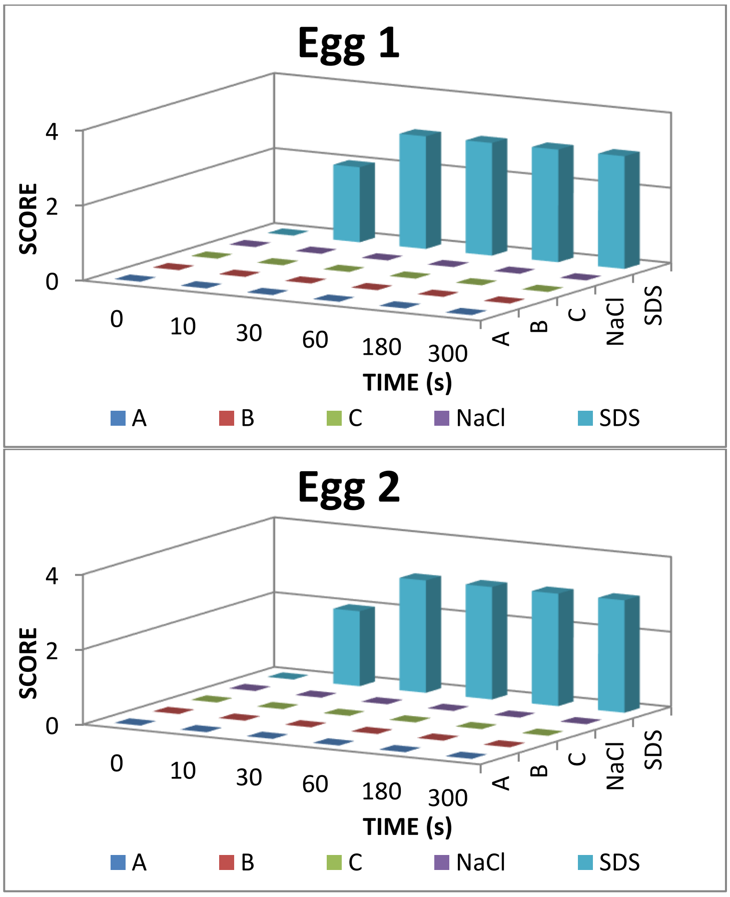

(c)

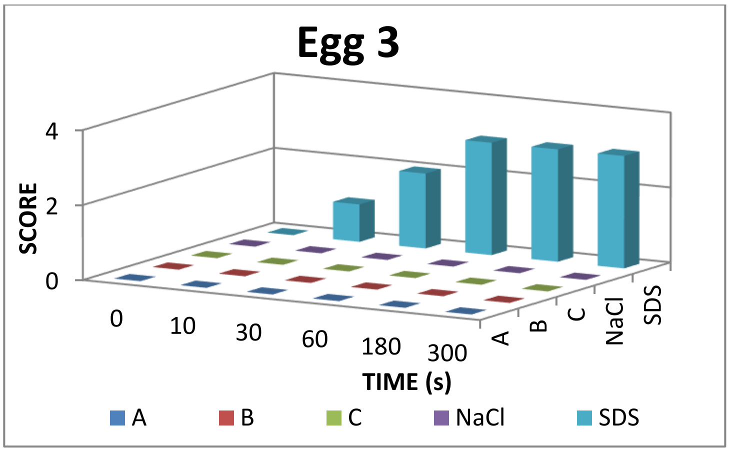

Figure S1. Hemorrhage score of (a) egg 1, (b) egg 2, and (c) egg 3. Egg 1 and egg 2 showed same score at the same time. Egg 3 had a different score for SDS at 10s and 30s with score of 1 and 2 while the other eggs had score of 2 and 3. Only SDS (positive control) had a hemorrhage in the egg's veins.

(a)

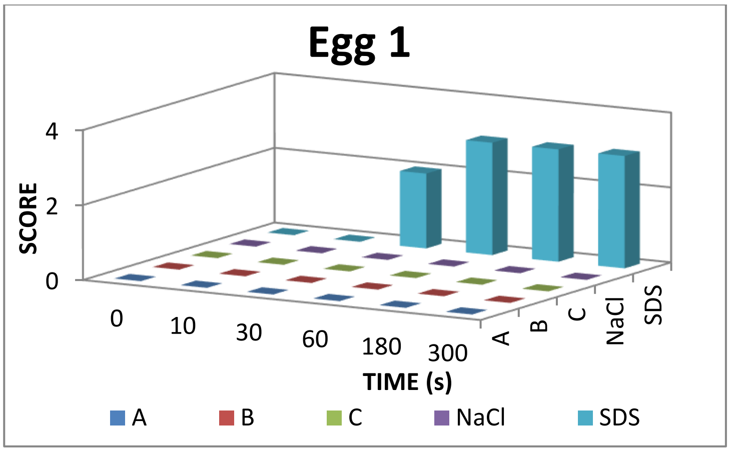

(b)

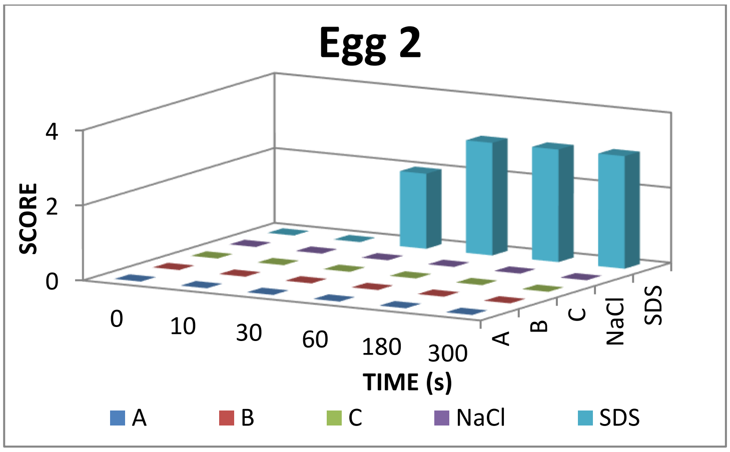

(c)

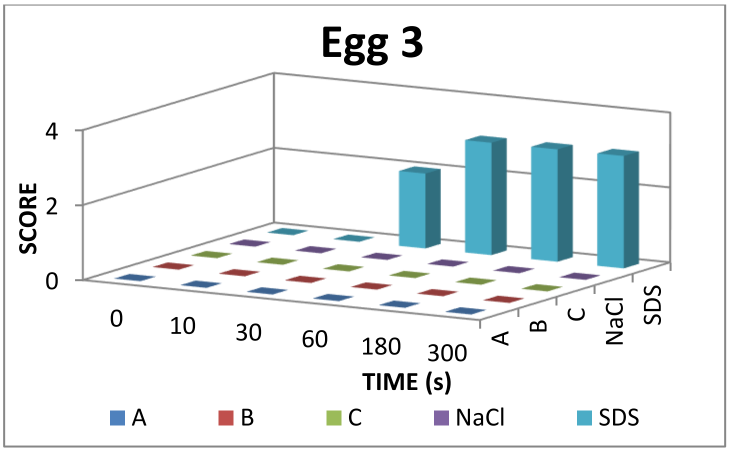

Figure S2. Lysis score of (a) egg 1, (b) egg 2 and (c) egg 3. All eggs showed same score at the same time. Only SDS (positive control) had a lysis in the egg's veins.

(a)

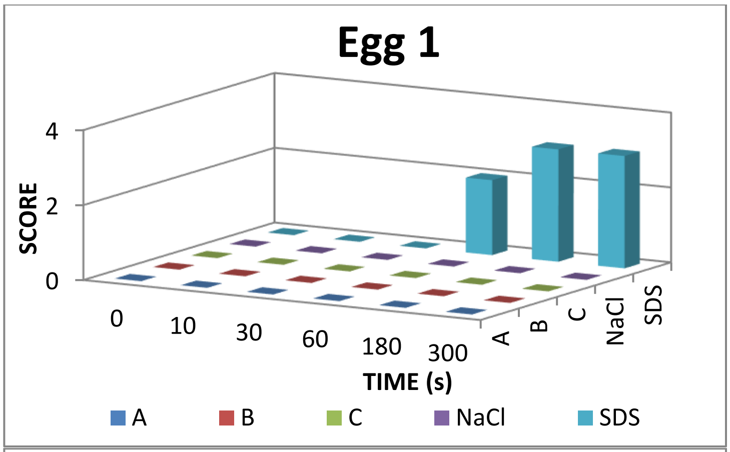

(b)

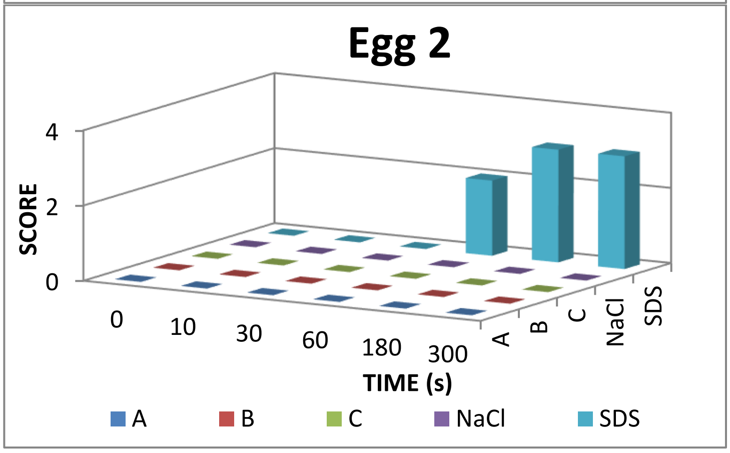

(c)

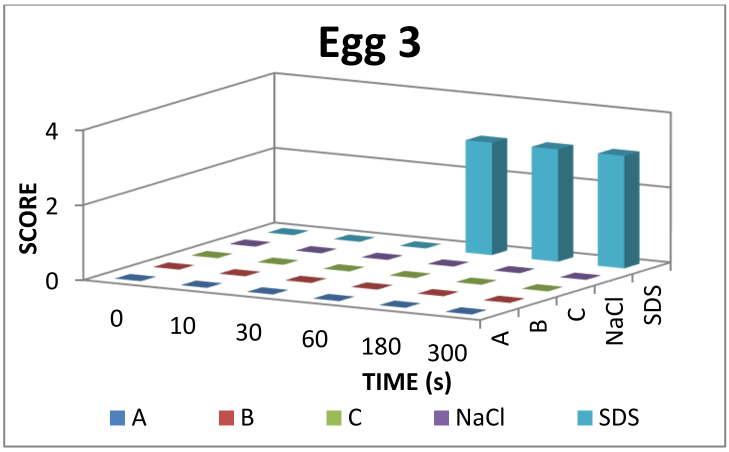

Figure S3. Coagulation score of (a) egg 1, (b) egg 2 and (c) egg 3. Egg 3 had a different score for SDS at 60s with score of 2 while the other eggs had score of 3. Only SDS (positive control) had a coagulation in the egg's veins.

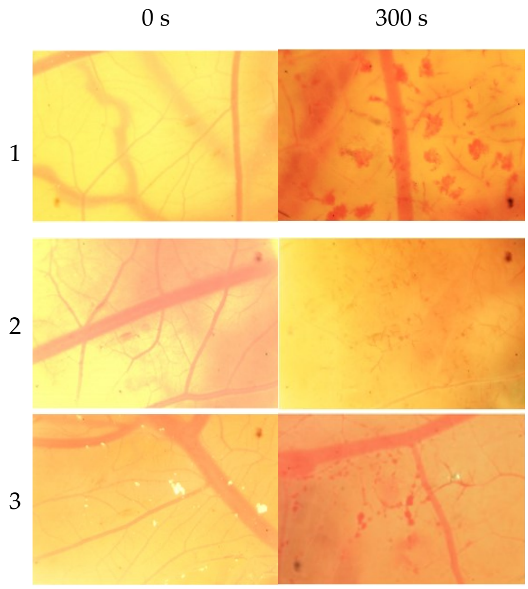

Figure S4. Vessels of positive control. The numbers 1, 2 and 3 represent egg 1, 2, and 3 for positive control group. All vessels of positive control group were damaged at 300s during HET-CAM test.

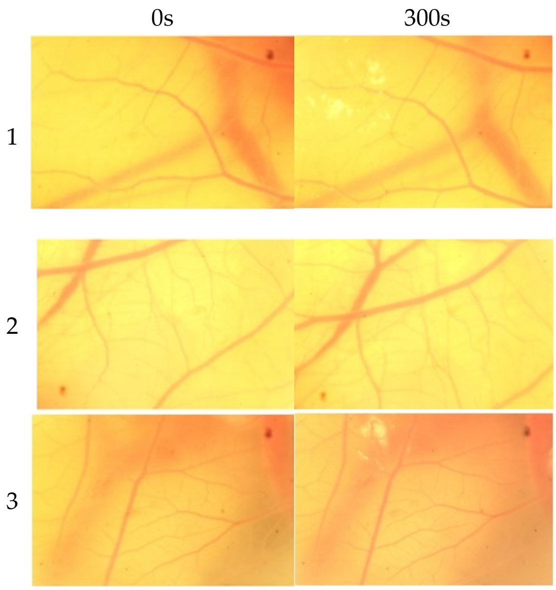

Figure S5. Vessels of sample A. The numbers 1, 2 and 3 represent egg 1, 2, and 3 for sample A group. All vessels of sample A group were not damaged during HET-CAM test.

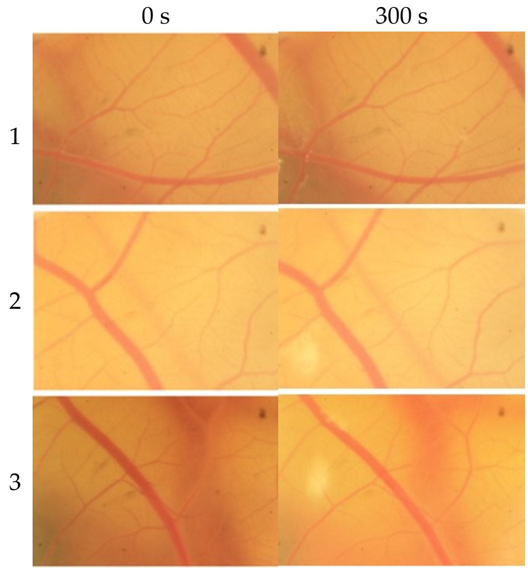

Figure S6. Vessels of sample B. The numbers 1, 2 and 3 represent egg 1, 2, and 3 for sample B group. All vessels of sample B group were not damaged during HET-CAM test.

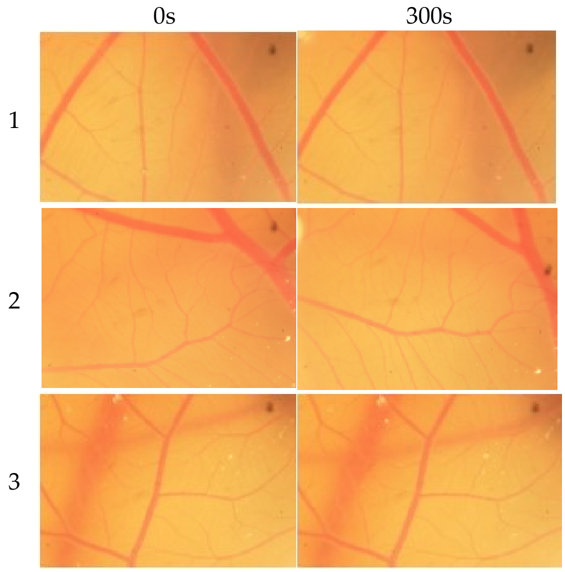

Figure S7. Vessels of sample C. The numbers 1, 2 and 3 represent egg 1, 2, and 3 for sample C group. All vessels of sample C group were not damaged during HET-CAM test.

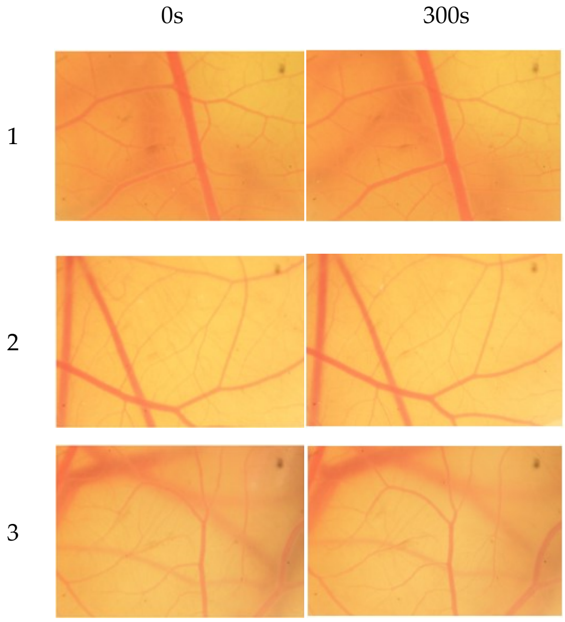

Figure S8. Vessels of the negative control. The numbers 1, 2 and 3 represent egg 1, 2, and 3 for negative control group. All vessels of sample C were not damaged during HET-CAM test.
